# Emerging Roles for Aberrant Astrocytic Calcium Signals in Parkinson’s Disease

**DOI:** 10.3389/fphys.2021.812212

**Published:** 2022-01-11

**Authors:** Eric A. Bancroft, Rahul Srinivasan

**Affiliations:** ^1^Department of Neuroscience and Experimental Therapeutics, Texas A&M University College of Medicine, Bryan, TX, United States; ^2^Texas A&M Institute for Neuroscience (TAMIN), College Station, TX, United States

**Keywords:** Parkinson’s disease, astrocytes, calcium, mitochondria, neurodegenenerative diseases

## Abstract

Astrocytes display a plethora of spontaneous Ca^2+^ signals that modulate vital functions of the central nervous system (CNS). This suggests that astrocytic Ca^2+^ signals also contribute to pathological processes in the CNS. In this context, the molecular mechanisms by which aberrant astrocytic Ca^2+^ signals trigger dopaminergic neuron loss during Parkinson’s disease (PD) are only beginning to emerge. Here, we provide an evidence-based perspective on potential mechanisms by which aberrant astrocytic Ca^2+^ signals can trigger dysfunction in three distinct compartments of the brain, *viz.*, neurons, microglia, and the blood brain barrier, thereby leading to PD. We envision that the coming decades will unravel novel mechanisms by which aberrant astrocytic Ca^2+^ signals contribute to PD and other neurodegenerative processes in the CNS.

## Introduction

Astrocytes are ubiquitous cells of the central nervous system (CNS) that outnumber neurons in many brain regions (von Bartheld et al., 2016). These cells are important players in governing neuronal function *via* mechanisms such as synaptic pruning, neurotransmitter clearance, and extracellular K^+^ buffering (Verkhratsky and Nedergaard, 2018). The critical role played by astrocytes in CNS function makes it vitally important to understand molecular mechanisms underlying bidirectional communication between astrocytes and neurons.

### Astrocytic Ca^2+^ Signals Are Important for Normal Central Nervous System Function

Unlike neurons, astrocytes are not electrically excitable, which has necessitated inquiry into the molecular machinery utilized by astrocytes to exert their functional effects on neurons and neural circuits. In this regard, studies utilizing genetically encoded calcium indicators (GECIs) such as GCaMPs have shown that astrocytes possess a plethora of spontaneous Ca^2+^ signals *in situ* and *in vivo*. Astrocytic Ca^2+^ signals respond to a variety of pharmacological and behavioral stimuli (Semyanov et al., 2020), and are observed in intracellular compartments such as the soma, thick proximal branches, and fine astrocytic processes (Srinivasan et al., 2015). In addition, astrocytic Ca^2+^ signals occur *via* Ca^2+^ release from distinct subcellular organelles such as the endoplasmic reticulum (ER; Okubo et al., 2019) and mitochondria (Huntington and Srinivasan, 2021), as well as extracellular Ca^2+^ sources (Srinivasan et al., 2015). At a subcellular level, the mechanisms governing astrocytic Ca^2+^ signals in the soma are distinct from those in peripheral processes (Verkhratsky et al., 2020). For example, Ca^2+^ signals in the soma and primary astrocytic processes occur due to metabotropic receptor activity, InsP_3_-mediated release of Ca^2+^ from the ER and store-operated Ca^2+^ entry. On the other hand, Ca^2+^ signals in fine astrocytic processes depend on mitochondrial Ca^2+^ fluxes, ionotropic channels such as transient receptor potential (TRP) and reversal of the Na^+^/Ca^2+^ exchanger, NCX. The presence of distinct compartments, mechanisms and sources for astrocytic Ca^2+^ signals strongly suggests that these signals modulate a diverse array of signaling pathways not only in the astrocytes themselves, but also in the neural circuits within which they reside.

Critical roles for spontaneous astrocytic Ca^2+^ signals in CNS function are bolstered by studies showing that these signals regulate the probability of neurotransmitter release (Covelo and Araque, 2018), long-term potentiation (Shigetomi et al., 2013; Arizono et al., 2020), maintenance of blood brain barrier (BBB) integrity (Heithoff et al., 2021), neurotransmitter clearance (Shigetomi et al., 2011; Haustein et al., 2014), and the synchronization and integration of neural activity (Sasaki et al., 2014; Pirttimaki et al., 2017; Deemyad et al., 2018). Since these processes are vitally important for normal CNS function, it is likely that a disruption in spontaneous astrocytic Ca^2+^ signaling is potentially pathological. In this regard, a particularly interesting question is how aberrant astrocytic Ca^2+^ signals could contribute to neurodegeneration.

### Aberrant Astrocytic Ca^2+^ Signaling and Parkinson’s Disease

Given their central role in brain function, it is not surprising that pathological alterations in astrocytes can accelerate the evolution of a variety of neurological diseases (Verkhratsky et al., 2017). Indeed, neurodegenerative diseases such as amyotrophic lateral sclerosis (ALS), and Alzheimer’s disease are characterized by distinct pathological changes in astrocytes. Examples of this include impaired glutamate uptake and death of motor neurons in ALS (Rossi et al., 2008; Valori et al., 2014) or reduced astrocyte coverage in Alzheimer’s disease, which results in synaptic deficiency and early cognitive dysfunction (Verkhratsky et al., 2016). Additionally, a recent study has shown that astrocytes derived from induced pluripotent stem cell (iPS) of Parkinson’s disease (PD) patients with a leucine rich repeat kinase 2 (LRRK2) mutation display fragmented mitochondrial morphology, atrophic cellular morphology, altered Ca^2+^ signaling and metabolic impairment (Ramos-Gonzalez et al., 2021). Together, these examples provide strong evidence for a central role of astrogliopathology in the evolution of neurodegenerative diseases.

Among the many known neurodegenerative disorders, PD is the second most common neurodegenerative disorder with no known cure (Poewe et al., 2017). PD is characterized by a progressive loss of substantia nigra pars compacta (SNc) dopaminergic (DA) neurons, and the onset of motor symptoms that include bradykinesia, resting tremors, postural instability, and muscle rigidity. Despite being labeled as a movement disorder, numerous non-motor symptoms are also observed during PD. These include sleep disturbances, constipation, anxiety, depression, and cognitive dysfunction (Poewe et al., 2017; Schapira et al., 2017). The complex clinical presentation of PD suggests a convergence of multiple mechanisms and cell types driving neurodegeneration. Most PD research, however, has focused on understanding pathological mechanisms that occur within the neurons themselves, without accounting for the role of astrocyte interactions with neurons, and other CNS cells during neurodegeneration. Consequently, neurocentric strategies have failed to result in the development of effective neuroprotective treatments for PD. In this context, we point to a central role for astrocytes, and more specifically, aberrant astrocytic Ca^2+^ signaling as an important contributing factor during the pathogenesis of PD.

**FIGURE 1:**
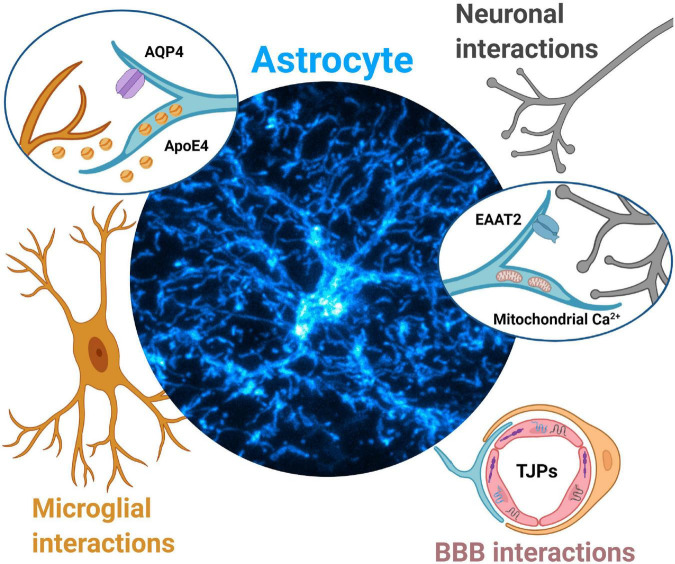
Aberrant astrocytic Ca^2+^ signals contribute to Parkinson’s disease pathology *via* multiple mechanisms. Neuronal interactions. Dopamine surges during early PD may dysregulate astrocytic Ca^2+^ signals and lead to EAAT2 internalization, leading to reduced glutamate clearance and initiation of excitotoxic cell death for dopaminergic midbrain neurons. Disruptions in astrocytic mitochondria Ca^2+^ signals (triggered from protein aggregates such as α-synuclein) lead to reduced ATP production, alteration of mitochondria-ER tethering, likely contributing to dopaminergic neurodegeneration. Microglial interactions. Aberrant astrocytic Ca^2+^ signals drive mislocalization of AQP4 channels in astrocytes. AQP4 deficiency in astrocytes is associated with increases in microglial activity and further secretion of inflammatory cytokines, ultimately contributing to dopaminergic neurodegeneration. Aberrant astrocytic Ca^2+^ signals may drive increased secretion of ApoE4 which leads to microglial reactivity, increased α-synuclein pathology and eventually dopaminergic neurodegeneration. Blood brain barrier (BBB) interactions. Aberrant Ca^2+^ signals in astrocyte endfeet may result in altered secretion of neurotrophic factors such as GDNF, leading to dysregulation of tight junction proteins (TJPs), compromised BBB integrity, and further contribute to dopaminergic neurodegeneration.

Given the rapidly emerging importance of astrocytes in PD (Booth et al., 2017), as well as an urgent and unmet need to develop effective neuroprotective treatments, this review presents a perspective on potential mechanisms by which aberrant astrocytic Ca^2+^ signals can trigger, and possibly sustain neurodegeneration during the development of PD. We amalgamate recent independent reports to provide an evidence-based rationale for the role of aberrant astrocytic Ca^2+^ signals in pathologically altering three distinct elements of the CNS during PD, *viz.* neurons, microglia, and the BBB (Figure 1).

## Aberrant Astrocytic Ca^2+^ Signals Can Cause Dysfunction in Dopaminergic Neurons

Protoplasmic astrocytes possess a bushy morphology with primary branches that give rise to very fine secondary branches, branchlets and leaflets (Moye et al., 2019; Zhou et al., 2019). Fine processes from each astrocyte, can contact upwards of 150,000 synapses in rodents and over a million synapses in humans (Bushong et al., 2002; Oberheim et al., 2009; Semyanov and Verkhratsky, 2021). Based on the morphological relationship of astrocytic processes with neuronal synapses, spontaneous Ca^2+^ signals in astrocytic processes are optimally positioned to modulate neuronal function. In addition, the intimate morphological as well as functional relationship between astrocytes and neurons suggests that abnormal changes in Ca^2+^ signals within astrocytic processes can alter neuronal function and initiate neurodegeneration. In the sections below, we gather evidence from recent independent studies to illustrate exemplar mechanisms by which abnormal changes in astrocytic Ca^2+^ signals can trigger, and even sustain the degeneration of SNc DA neurons.

### Excitatory Amino Acid Transporter 2

Excess extracellular glutamate is a major mechanism for neurodegeneration (Ambrosi et al., 2014; Lewerenz and Maher, 2015). This can occur *via* mechanisms such as glutamate-mediated excitotoxicity (Lewerenz and Maher, 2015), oxidative glutamate toxicity (Schubert and Piasecki, 2001; Wang et al., 2020), and immunoexcitotoxicity (Blaylock, 2017). Astrocytes play a major role in neurotransmitter clearance (Eulenburg and Gomeza, 2010) and specifically, glutamate clearance *via* astrocytic glutamate transporters such as excitatory amino acid transporter 2 (EAAT2) (Lehre and Danbolt, 1998). Therefore, any reduction in astrocytic EAAT2 expression would result in abnormal levels of extracellular glutamate and neurodegeneration. Indeed, reductions in astrocytic EAAT2 expression are observed in multiple neurodegenerative diseases such as amyotrophic lateral sclerosis, Alzheimer’s disease and Huntington’s disease (Bruijn et al., 1997; Tong et al., 2014; Sharma et al., 2019). With regard to PD, two recent pieces of evidence are particularly relevant: (**i**) The targeted knockdown of EAAT2 in astrocytes causes degeneration of SNc DA neurons in a mouse model of PD (Zhang et al., 2020) and (**ii**) Exposure of rodents to the PD toxin 6-hydroxydopamine (6-OHDA) causes a downregulation of EAAT2 (Chotibut et al., 2017).

A recent study shows that EAAT2 internalization from the surface of astrocytes increases in a Ca^2+^-dependent manner (Ibanez et al., 2019). Specifically, Ca^2+^ influx *via* the NCX sodium/calcium exchanger in response to increases in extracellular glutamate results in EAAT2 internalization. In a broader sense, one could infer that increased Ca^2+^ influx within astrocytes due to abnormal increases in extracellular neurotransmitters could result in EAAT2 internalization. We rationalize that a surge in striatal dopamine levels during early PD, as seen in the Thy1-α-synuclein mouse model of PD (Lam et al., 2011) can cause a downregulation of EAAT2 in striatal astrocytes. A recent study by Adermark et al. (2021) showed that pre-treatment of striatal brain slices with the D2 dopamine receptor agonist, sulpiride prevented synaptic depression induced by the EAAT2 blocker, TFB-TBOA. These data suggest a rapid downregulation of EAAT2 function in striatal astrocytes due to an abnormal activation of striatal D2 receptors. In addition, studies have shown that synaptically released dopamine increases Ca^2+^ events in striatal astrocytes (Corkrum et al., 2020), and the activation of D2 receptors in ventral midbrain astrocytes causes a downregulation of EAAT2 expression (Xin et al., 2019).

When taken together, these studies point to aberrant dopamine-mediated Ca^2+^ signals in astrocytic processes as a potential mechanism for EAAT2 downregulation in astrocytes leading to excess extracellular glutamate and consequently, neurodegeneration.

### Astrocytic Mitochondria

Recent studies have shown that astrocytic processes contain mitochondria (Derouiche et al., 2015; Agarwal et al., 2017; Huntington and Srinivasan, 2021) and that mitochondria in fine astrocytic processes are closely associated with Ca^2+^ signals in their vicinity (Agarwal et al., 2017). Interestingly, the Ca^2+^ signals associated with astrocytic mitochondria are abnormally increased in a mouse model of amyotrophic lateral sclerosis expressing a mutant form of superoxide dismutase (SOD G93A) (Agarwal et al., 2017), suggesting a role for abnormal mitochondrial Ca^2+^ signaling in fine astrocytic processes during neurodegeneration. In addition, we have shown that mitochondria in astrocytic processes display spontaneous Ca^2+^ influx with dual responses to neurotransmitter agonists, a dependency on ER Ca^2+^, and the absence of functional mitochondrial uniporters (MCU; Huntington and Srinivasan, 2021), suggesting that astrocytic mitochondria possess unique functional properties that optimally cater to the extensive energy needs of DA neurons. A significant proportion (∼25%) of energy demands in the CNS are met by astrocytes (van Hall et al., 2009), and spontaneous astrocytic mitochondrial Ca^2+^ signals derived from the endoplasmic reticulum (ER) activate mitochondrial dehydrogenases in order to generate the co-factors required for oxidative phosphorylation and ATP generation (Cardenas et al., 2010). Disruption of Ca^2+^ signals in astrocytic mitochondria could therefore be a harbinger for neurodegeneration.

Given the important role of α-synuclein (α-syn) in the pathogenesis of PD, it is pertinent to discuss aberrant Ca^2+^ signals in mitochondria within astrocytic processes as it relates to α-syn pathology. Recent studies have shown that astrocytes readily take up extracellular α-syn aggregates, and that α-syn can damage astrocytic mitochondria (Lindstrom et al., 2017), as well as alter molecular tethering between the mitochondria and ER, resulting in disrupted Ca^2+^ homeostasis (Paillusson et al., 2017). Together, these reports suggest that pathological forms of α-syn can bind to astrocytic mitochondria, which could alter mitochondrial Ca^2+^ signaling in astrocytes, thereby causing a significant reduction in ATP generation and DA neuron loss. It is also important to note that astrocytes are coupled *via* gap junctions (Fujii et al., 2017). Therefore, pathological changes in mitochondrial Ca^2+^ signals in just a few astrocytes can affect larger populations of astrocytes within neural structures, thus magnifying the effects of aberrant astrocytic mitochondrial Ca^2+^ signaling on brain function and neurodegeneration.

## Aberrant Astrocytic Ca^2+^ Signals Can Activate Microglia

Microglia are classically viewed as the immune surveillance cells of the brain, with functions that include phagocytosis and synaptic pruning (Li and Barres, 2018; Bohlen et al., 2019; Bartels et al., 2020). In the context of DA neuron loss in PD, microglial reactivity is a strong indicator of neuroinflammation and ongoing neuropathology. There is evidence for microglial activation in clinical PD (McGeer et al., 1988; Gerhard et al., 2006; Bartels et al., 2010; Stokholm et al., 2017), as well as in rodent (Czlonkowska et al., 1996; Wu et al., 2002; Sanchez-Guajardo et al., 2010; Hoenen et al., 2016) and non-human primate models of parkinsonism (McGeer et al., 2003; Barcia et al., 2004; Kanaan et al., 2008; Barkholt et al., 2012). There are potential mechanisms by which aberrant Ca^2+^ signals in astrocytes could play a role in initiating microglial activation during PD. In the sections below, we present two potential scenarios in which aberrant changes in spontaneous astrocytic Ca^2+^ signals could lead to abnormal crosstalk between astrocytes and microglia, thus accelerating neuronal loss in PD.

### Aquaporin 4

Aquaporin 4 (AQP4) is a tetrameric water channel, abundantly expressed in astrocytes (Hubbard et al., 2015; Tham et al., 2016). Emerging evidence suggests a role for AQP4 dysfunction in PD. Studies supporting this idea include findings that: (**i**) Humans with Lewy body pathology in the neocortex demonstrate a negative correlation between AQP4 expressing astrocytes and α-synuclein aggregates, such that astrocytes with AQP4 expression do not appear in areas with abnormal α-synuclein expression (Hoshi et al., 2017), (**ii**) Exposure of AQP4 knockout (KO) mice to the PD toxin, MPTP causes an increase in the susceptibility of SNc DA neurons to degeneration (Fan et al., 2008), (**iii**) AQP4 KO mice show diminished differences between ventral tegmental area (VTA) and SNc DA neurons in their susceptibility to MPTP-induced neurodegeneration (Zhang et al., 2016), and (**iv**) AQP4 knockout mice display significant increases in microglial reactivity following exposure to MPTP when compared to wildtype littermates. In this case, the study also shows that the increase in microglial reactivity occurs due to secretion of neuroinflammatory molecules such as interleukin 1β (IL1β) and tumor necrosis factor α (TNFα; Sun et al., 2016). When taken together, these studies converge on the idea that a functional deficiency of AQP4 in astrocytes can result in microglial activation with a consequent increase in the secretion of neuroinflammatory molecules by activated microglia, in turn resulting in the loss of DA neurons.

As is the case for any channel, the ability of AQP4 to allow passage of water molecules through its pore requires precise localization at the plasma membrane. In this regard, studies show that AQP4 depends on Ca^2+^ for localization to the plasma membrane (Salman et al., 2017; Kitchen et al., 2020), and that rapid translocation of AQP4 to the plasma membrane depends on Ca^2+^ signals. Furthermore, a recent study has utilized STORM-based superresolution microscopy to show that AQP4 channels cluster in very specific patterns at astrocytic endfeet (Smith and Verkman, 2015). Thus, there exists an intricate relationship between Ca^2+^ signaling and the normal functional localization of AQP4 in astrocytes. Based on these data, one can infer that pathological changes in spontaneous astrocytic Ca^2+^ signals will result in the mislocalization and functional deficit of astrocytic AQP4, leading to microglial activation and neuroinflammation in the brain.

### Apolipoprotein E

An allelic variant of the apolipoprotein E (ApoE) gene, ApoE4 significantly increases the risk for Alzheimer’s disease (AD; Lambert et al., 2013; Liu et al., 2013; Yamazaki et al., 2019). In the case of PD, a recent study created ApoE locus-targeted ApoE4 replacement mice, and utilized these mice to show that ApoE4 increases α-synuclein pathology, worsens behavioral deficits, and accelerates astrogliosis (Zhao et al., 2020). This study also showed that ApoE4 increases α-synuclein pathology in PD patients.

In the CNS, astrocytes are a major reservoir for ApoE (Sun et al., 1998; Xu et al., 2006), and ApoE4 secretion occurs in a Ca^2+^-dependent manner (Kockx et al., 2007). Thus, any pathological alteration in the kinetics of astrocytic Ca^2+^ signals can alter the secretion of ApoE4 from astrocytes. Based on this rationale, increases in spontaneous astrocyte Ca^2+^ signal amplitudes as seen in reactive astrocytes (Shigetomi et al., 2019) could increase ApoE4 secretion by astrocytes, leading to microglial activation (Maezawa et al., 2006; Vitek et al., 2009), increased α-synuclein uptake by microglia (Choi et al., 2020), the formation of toxic α-synuclein aggregates (Davis et al., 2020) and neurodegeneration. Although we do not yet know what may initiate aberrant Ca^2+^ signaling in astrocytes, abnormal α-synuclein uptake by astrocytes could disrupt Ca^2+^ homeostasis, and is therefore a likely candidate for triggering aberrant Ca^2+^ signals in SNc astrocytes during PD.

## Aberrant Astrocytic Endfoot Ca^2+^ Signals and Blood Brain Barrier Integrity

The BBB is an important protective barrier that allows selective passage of molecules into the brain parenchyma. Abnormal increases in BBB permeability can allow the passage of environmental toxins into the midbrain, thereby accelerating DA neuron loss. This view is supported by the epidemiological finding that pesticide exposure is associated with an increased incidence of PD in farmers (Freire and Koifman, 2012). In this context, a histological study of striatal brain sections from PD patients has shown abnormal extravasation of erythrocytes, as well as an increase in extravascular serum proteins such as fibrin and hemoglobin into striatal parenchyma, suggesting a loss of BBB integrity during PD (Gray and Woulfe, 2015). Another recent study used dynamic contrast enhanced magnetic resonance imaging in 49 PD patients to show significantly higher BBB leakage in posterior white matter regions of PD patients compared to healthy controls (Al-Bachari et al., 2020). These studies suggest that a loss of BBB integrity is likely involved in the pathogenesis of clinical PD.

The emerging evidence for a compromised BBB in PD patients motivates inquiry into whether or not astrocytes contribute to the maintenance of BBB integrity. In this regard, a recent study has utilized GLAST Cre/ERT2 mice driving the expression of the diphtheria toxin in astrocytes to ablate astrocytes in sparse regions of blood vessels. This study showed an extravasation of cadaverine from blood vessels following toxin-induced astrocyte ablation in mice (Heithoff et al., 2021), which strongly suggests that astrocytic endfeet do indeed play a central role in maintaining the physical integrity of the BBB.

Together, the findings described above lead to the important question of whether or not disruptions in Ca^2+^ signals in astrocytic endfeet could compromise the established dependence of tight junction proteins (TJPs) on Ca^2+^ (Stuart et al., 1994; Brown and Davis, 2002), thereby altering BBB integrity. Although there is currently no clear evidence for a causative role of aberrant endfoot Ca^2+^ signals in altering TJP biology in PD, the use of new imaging modalities such as multiphoton microscopes in combination with astrocyte-specific transgenic mice (Srinivasan et al., 2016) and genetically encoded Ca^2+^ sensors in astrocytes should enable an understanding of the role of aberrant astrocytic endfoot Ca^2+^ signals in TJP and BBB function during PD.

## Conclusion

In this perspective review, we discuss potential pathological mechanisms during PD in which aberrant astrocytic Ca^2+^ signals cause either neuronal dysfunction, microglial activation, or a loss of BBB integrity (Figure 1). Although we do not discuss what triggers abnormal Ca^2+^ signals in astrocytes during PD in the first place, molecules such as ApoE4 and α-synuclein likely initiate abnormal Ca^2+^ signaling in astrocytes *via* multiple and distinct mechanisms. It is therefore reasonable to hypothesize that once they are initiated, abnormal astrocytic Ca^2+^ signals cause further abnormalities in ApoE4 or α-synuclein, thereby setting up a vicious feedback loop between aberrant astrocytic Ca^2+^ signaling and ApoE4 or α-synuclein pathology in PD.

An additional point to note is that neurons, microglia and the BBB are also capable of directly interacting with each other, which would result in a complex network of multi-tiered pathological interactions. Based on this view, we predict that the coming decades will unravel specific mechanisms by which aberrant astrocytic Ca^2+^ signals modulate multi-tiered interactions between these seemingly distinct CNS compartments, eventually leading to neurodegeneration.

## Author Contributions

RS conceived and wrote the manuscript. RS and EB compiled and edited the manuscript. EB created the figures with input from RS. Both authors contributed to the article and approved the submitted version.

## Conflict of Interest

The authors declare that the research was conducted in the absence of any commercial or financial relationships that could be construed as a potential conflict of interest.

## Publisher’s Note

All claims expressed in this article are solely those of the authors and do not necessarily represent those of their affiliated organizations, or those of the publisher, the editors and the reviewers. Any product that may be evaluated in this article, or claim that may be made by its manufacturer, is not guaranteed or endorsed by the publisher.
